# A case report: Unilateral biportal endoscopic revision for adjacent segmental disease: Case presentations and literature review

**DOI:** 10.1097/MD.0000000000035466

**Published:** 2023-10-06

**Authors:** Chengyue Zhu, Yujun Zhang, Susu Sun, Rongxue Shao, Jiaming Liang, Wei Cheng, Hao Pan, Wei Zhang

**Affiliations:** a Department of Orthopaedics, Hangzhou Traditional Chinese Medicine Hospital Affiliated to Zhejiang Chinese Medical University, Hangzhou, China; b Hangzhou School of Clinical Medicine, Zhejiang Chinese Medical University, Hangzhou, China.

**Keywords:** adjacent segmental disease, lumbar stenosis, minimally invasive spine surgery, revision surgery, unilateral biportal endoscopy

## Abstract

**Rationale:**

Biportal endoscopic revision surgery for adjacent segmental disease (ASD) after lumbar arthrodesis is seldomly reported. Herein, we present 3 cases of ASD with radiculopathy wherein satisfactory results were obtained using unilateral biportal endoscopic (UBE) decompression.

**Patient concerns:**

Case 1 was of a 56-year-old male who presented with a chief complaint of Intermittent claudication since 2-year. Case 2 involved a 78-year-old female who was admitted to the hospital with a chief complaint of radiating pain and weakness in the left leg for at least 1 year. Case 3 was a 67-year-old woman who visited our hospital because of radiating leg pain for 5 months. All the cases had a history of L4 to L5 lumbar interbody fusion surgery.

**Diagnoses:**

Computed tomography and magnetic resonance imaging showed the spinal epidural lipomatosis at the L3 to L4 level in case 1, the up-migrated lumbar disc herniation at L3 to L4 level in case 2 and unilateral foraminal stenosis at the L5 to S1 level in case 3.

**Interventions:**

Under UBE guidance, the ipsilateral approach was used to treat adjacent lumbar stenosis caused by spinal epidural lipomatosis. The contralateral approach was used to remove the up-migrated herniated disc. The paraspinal approach was applied to decompress the foraminal stenosis.

**Outcomes:**

Postoperative parameters were improved clinically, and nerve roots were decompressed radiologically. No complications were developed.

**Lessons:**

UBE revision surgery showed a favorable clinical and radiological result without complications and may be a safe and effective alternative technique for ASD.

## 1. Introduction

With the wide application of lumbar arthrodesis for the treatment of lumbar degenerative disease, the prevalence of adjacent segmental disease (ASD) has increased accordingly.^[1]^ The common risk factors for ASD include the number of fused levels, laminectomy levels, lumbar sagittal alignment, and use of cages.^[2,3]^ Symptomatic ASDs, which are refractory to conservative treatment, always need surgical intervention. Previous studies have reported the incidence of reoperation ranging from 4.1% to 38%.^[4,5]^ Conventional open extension of fusion is the main modality for ASD revision; however, the access-related complications, such as infections and durotomy, cannot be underestimated,^[6,7]^ and the rate of revision surgery following reoperation ranges from 4.5% to 23.1% at the final follow-up.^[8]^ Thus, minimally invasive spine surgery has been proposed to avoid complications. McGrath^[9]^ has reported an early experience in endoscopic foraminal decompression of the fused segments and resection of displaced interbody cages. Li,^[10]^ using the same mono-portal technique, has reported similar outcomes for the treatment of stable ASD compared with extended posterior lumbar interbody fusion (PLIF). Unilateral biportal endoscopy (UBE), which has many advantages, including flexible manipulation, wide surgical field, and high efficiency over mono-portal endoscopy,^[11–13]^ will theoretically achieve a better clinical result. Herein, to the best of our knowledge, we first describe a case series and UBE surgical technique for ASD treatment.

## 2. Materials and methods

This study analyzed 3 patients who underwent UBE revision surgery after PLIF. Clinical data were reviewed, including age, sex, symptoms, imaging, surgical approaches, visual analog scale (VAS), neurological intermittent claudication (NIC), and Oswestry disability index. This study was approved by the Institutional Review Board of Hangzhou Traditional Chinese Medicine Hospital, Zhejiang Chinese Medical University (no. 202007102020000214122), and all patients provided their informed consent.

All the patients were placed on a radiolucent table in the prone position with mild flexion of the lumbar spine, and all the revision procedures were performed under general anesthesia. Curettes, osteotome, punches, radiofrequency, and highspeed burr were prepared before operations.

## 3. Case reports

### 3.1. Case 1

**Figure 1.**
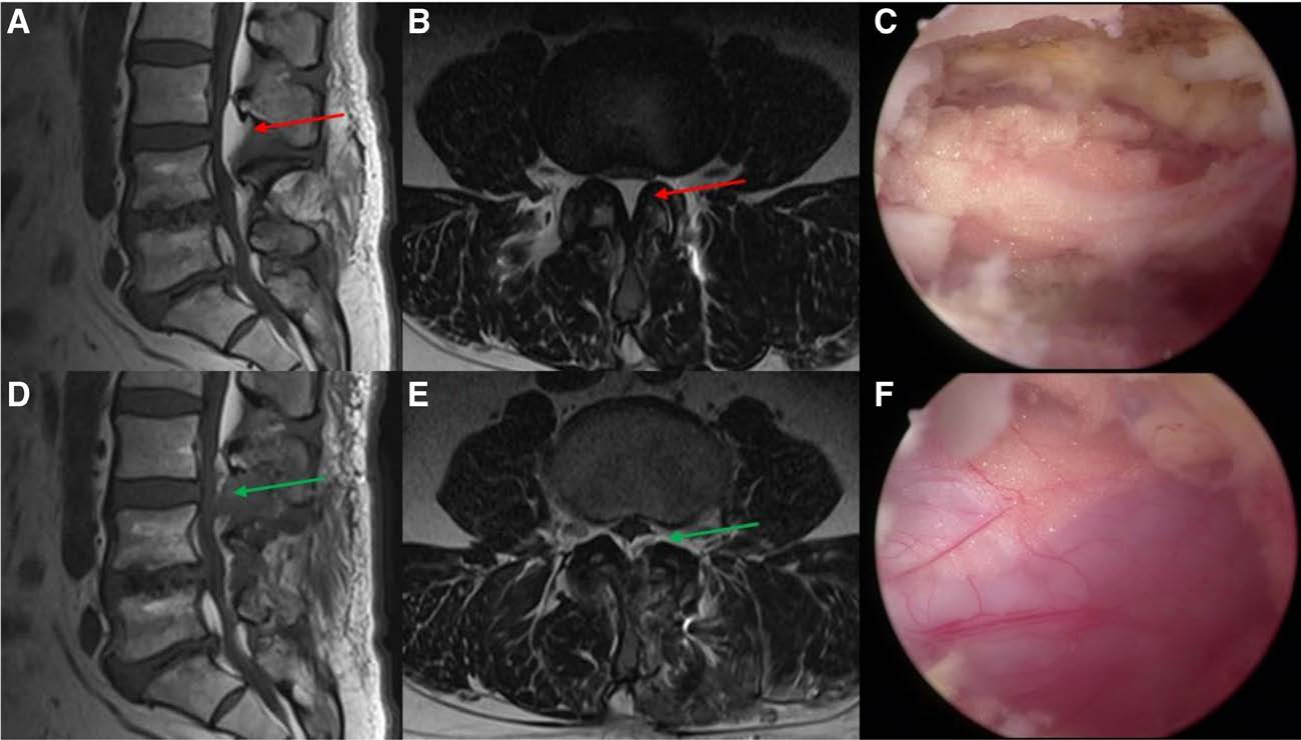
(A, B) Sagittal and axial MRI showed fat deposition (red arrow) compressing the dural sac. (C) Endoscopic image showing fat deposition in the spinal canal after laminotomy and flavectomy. (D, E) Sagittal and axial MRI showed a decompressed dural sac (green arrow) (F) The dural sac was completely decompressed after the fatty tissue was removed. MRI = magnetic resonance imaging.

A 56-year-old man with hypertension, diabetes, and a body mass index of 31 kg/m^2^ presented with a 2-year history of NIC 10 years after undergoing L4 to L5 PLIF. The patient complained of moderate back and leg pain and chronic sensory changes in both legs. Although he could walk, knee extension and ankle and great toe dorsiflexors showed weakness (grade 4 muscle strength). The symptoms had not ameliorated significantly in the past 3 months, during which the patient received conventional conservative treatment. Magnetic resonance imaging (MRI) and computed tomography (CT) revealed spinal epidural lipomatosis at the L3 to L4 level (Fig. 1). Based on the radiological findings and unbearable symptoms, surgery was indicated.

After sterilization with povidone-iodine, the operative level was confirmed under fluoroscopic guidance, and incision sites were marked using an antiseptic pen. Two incisions were made along the line running through the medial border of the L3 to L4 pedicle, 1.5 cm above and below the intersection of the spinous process and lamina. The cranial incision was used as an endoscopic portal, whereas the caudal incision was used as the working portal. The paraspinal muscles were detached using a blunt dissector, and the soft tissue on the bone surface was peeled off using a radiofrequency probe. The inferior lamina of the superior vertebra was partially removed until the superior edge of the ligamentum flavum (LF) was exposed, followed by LF dissection and complete resection to the superior edge of the superior lamina of the inferior vertebra. The proliferated fat was identified and removed using a Kerrison punch and pituitary forceps. At the end of the procedure, dura sac pulsation was confirmed, and a drainage catheter was inserted (see Video 1, Supplemental Digital Content Video 1, http://links.lww.com/MD/K152, which demonstrates biportal endoscopic removal of excessive epidural fat). The operation time was 45 minutes. Postoperatively, the patient had complete relief of back and leg pain, and successful removal of the epidural proliferated fat was confirmed on MRI (Fig. 1). The patient was discharged 2 days postoperatively. He could perform all his daily activities and remained asymptomatic 12 months later.

### 3.2. Case 2

A woman aged 78 years with hypertension underwent a L4 to L5 PLIF 2 years ago, and her body mass index was 18 kg/m^2^. She complained of radiating pain and weakness in the left leg for at least 1 year that deteriorated significantly in the past 3 months. The VAS score of leg pain was 5, and the pain was localized in the left anterior thigh area. Other physical examinations showed slight back pain (VAS 2), hypesthesia, and attenuation of tendon reflex in the left lower limb. On MRI and axial CT scan, the up-migrated disc herniation was observed at L3 to L4 level, and both the traversing and exiting nerve roots were compressed. The patient expressed strong opposition to extended fusion surgery and agreed to a reoperation using the UBE technique.

**Figure 2.**
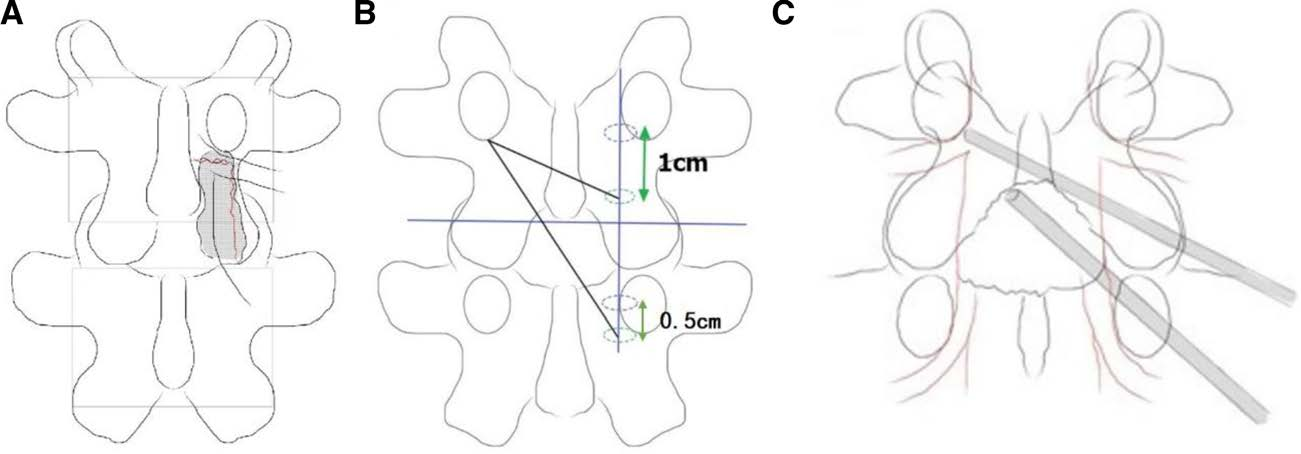
(A) UBE discectomy for up-migrated disc herniation via the ipsilateral approach can easily damage the isthmus. (B) The ideal “right to left” portals for the UBE contralateral sublaminar approach. (C) Schematic representation of the operation. UBE = unilateral biportal endoscopy.

Conventionally, the viewing and working portals were made over the medial wall of the pedicles on the anteroposterior view, but to remove the up-migrated disc and decompress the exiting root on the contralateral side, the viewing and working portals were moved by 0.5 cm and 1 cm downward, respectively (Fig. 2).

The endoscope and radiofrequency (RF) probe were triangulated toward each other until the tip ends met at the junction of the spinous process and right-sided lamina of L3, initial working space was created after the soft tissue on the surface of the lamina was dissected using RF. Partial ipsilateral laminotomy was performed to expose the caudal and cranial entheses of LF. The base of the spinous process was generously removed by a curved sheathed drill and Kerrison punches; then, the “V” shape parts between the ipsilateral and contralateral LFs were exposed. Contralateral LF was detached from the ventral surface of the lamina with a probe, and contralateral sublaminoplasty was performed until contralateral LF was liberated.

A ball-tip probe could be used to separate the LF and the underlying dura gently, and the ipsilateral LF was partially removed for better operation field of the dura mater in the longitudinal direction. A straight Kerrison punch was used to remove contralateral LF in a superolateral direction with a piecemeal method. A partial resection maneuver of the contralateral superior articular process (SAP) of L4 was performed with a curved Kerrison punch, and the LF in the foraminal area was simultaneously removed, followed by the visualization of the up-migrated herniated disc between the shoulder region of traversing root and axillar area of exiting root.

**Figure 3.**
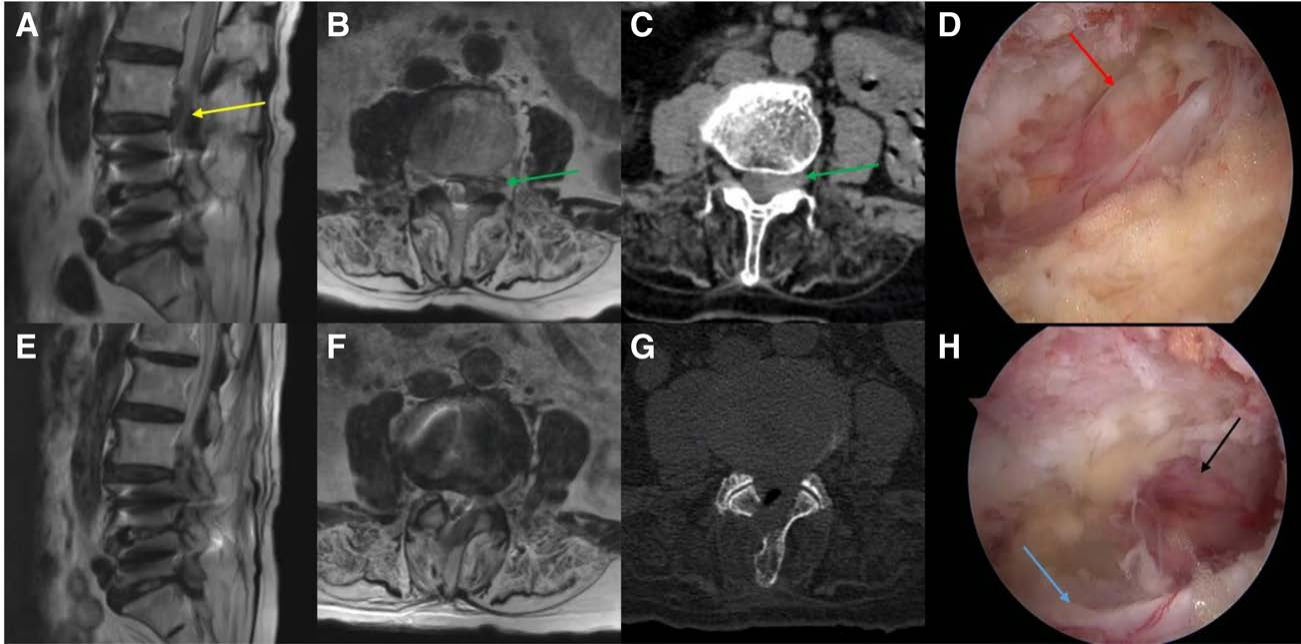
(A) Preoperative sagittal MRI showed an up-migrated herniated disc at the L3–L4 level (yellow arrow), (B, C) axial MRI and CT showed that the herniated disc was partially intruding in the foraminal area (green arrow), (D) the up-migrated herniated disc (red arrow) was exposed, (E, F) Postoperative sagittal and axial MRI revealed that the herniated disc was removed, and nerve roots were decompressed, (G) axial CT showed that the facet joint was less involved, and (H) the exiting (black arrow) and traversing (blue arrow) nerve roots were completely decompressed. CT = computed tomography, MRI = magnetic resonance imaging.

A small tip RF probe was used to incise the membrane containing the herniated disc, and angled pituitary forceps of different sizes were used to remove the ruptured discs, ensuring no injury to the dura mater and contralateral nerve roots. Repeated exploration was carefully performed with a blunt hook for any remnant disc particles. After confirming the contralateral traversing and exiting nerve roots were completely decompressed (Fig. 3), we stopped the irrigation to identify the bleeding area. Then, hemostasis was performed with the RF probe and bone wax. Finally, a drainage tube was inserted through the working portal under the guidance of endoscopy (see Video 2, Supplemental Digital Content Video 2, http://links.lww.com/MD/K153, which shows the upwards migration of L3 to L4 lumbar disc herniation (LDH) treated via a contralateral approach using biportal endoscopic discectomy). After the discectomy, NIC improved from 10 minutes to 60 minutes. The modified MacNab criteria score was graded as excellent, and no complications were observed.

### 3.3. Case 3

A 67-year-old woman who underwent PLIF 2 years ago visited our hospital because of radiating leg pain and NIC lasting <10 minutes for 5 months. The pain radiated to the left L5 dermatome. The VAS score was 6, and she developed subjective weakness in the left ankle. Imaging revealed unilateral foraminal stenosis below the fusion level, and a paraspinal approach was applied to decompress the exiting root of L5.

**Figure 4.**
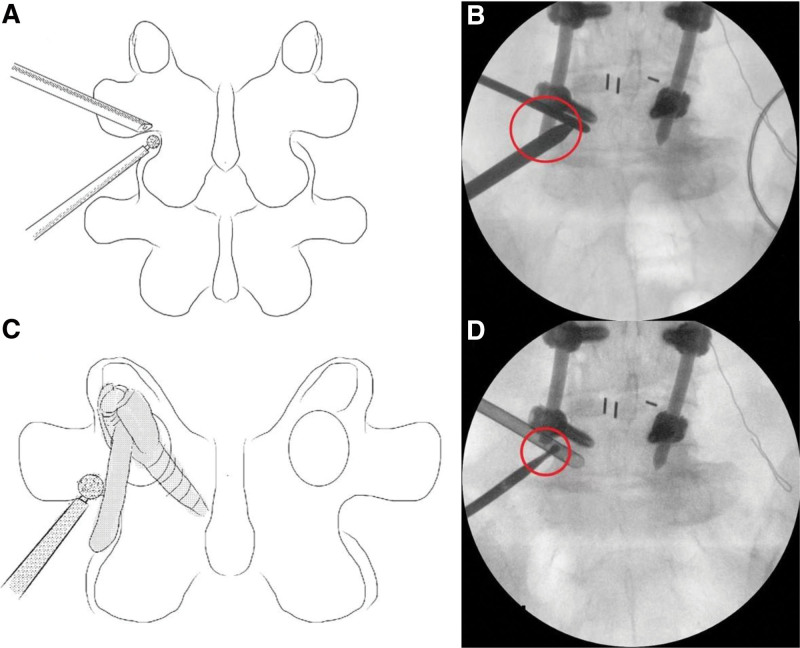
(A) The landing mark of the UBE paraspinal approach is the lateral wall of the isthmus. (B) The landing mark was occupied by the distal end of the rod (red circle). (C) Schematic representation of drilling the rod. (D) The distal end of the rod was removed (red circle). UBE = unilateral biportal endoscopy.

**Figure 5.**
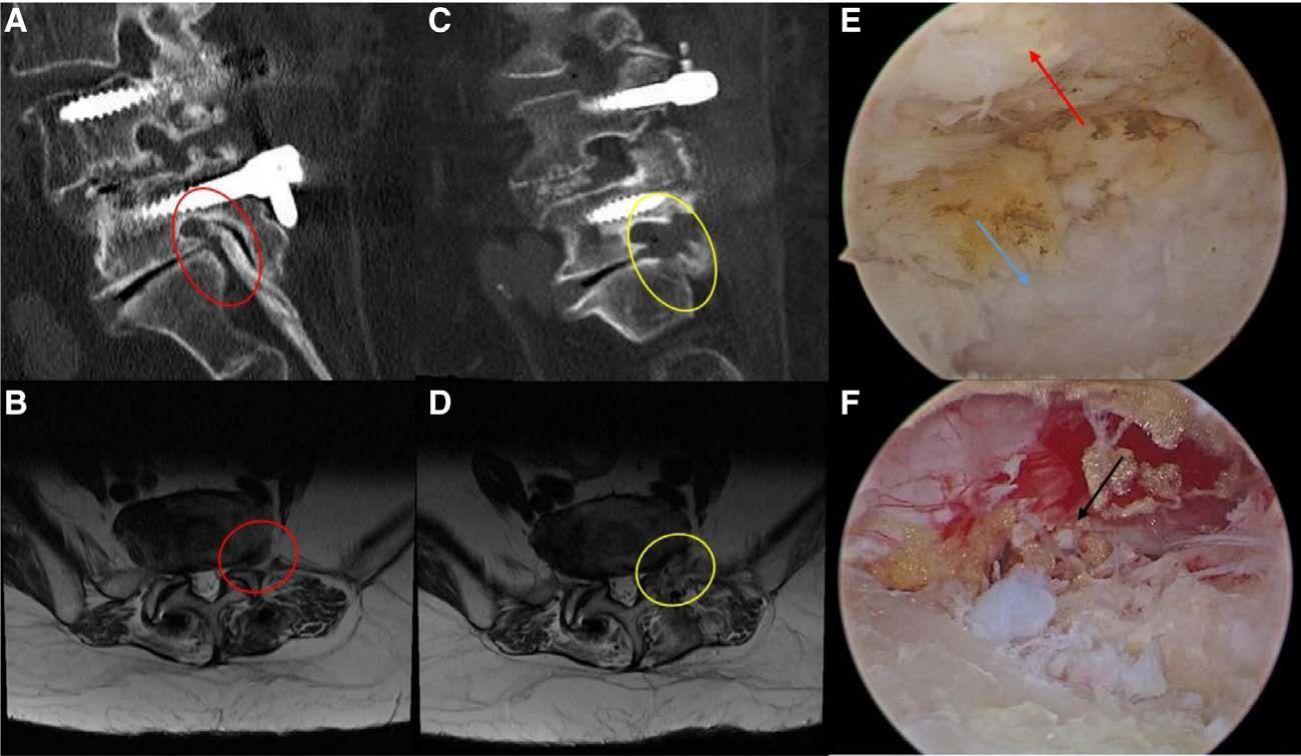
(A, B) Preoperative sagittal CT and axial MRI showed the left foraminal stenosis at the L5–S1 level (red circle), (C, D) foraminotomy was performed, and the exiting nerve root was decompressed (yellow circle), (E) endoscopic view of foraminal stenosis: LF in the foramen (red arrow), SAP (blue arrow), and (F) endoscopic image showed the foramen after decompression. The black arrow indicates the exiting nerve root. CT = computed tomography, LF = ligamentum flavum, MRI = magnetic resonance imaging, SAP = superior articular process.

Two incisions were made 2 cm lateral to the L5 to S1 pedicle lateral margin, 1.5 cm above and below the isthmus. The incision on the left-hand side served as a viewing portal, and the other on the right-hand side served as a working portal. The approach was blocked by the left-sided rod, and we had to remove the tip of the rod with a 4-mm highspeed diamond burr (Fig. 4). The soft tissue around the tip of SAP of S1 and isthmus of L5 was detached by RF. The tip of SAP was removed using a chisel and burr; then, the LF in the foraminal area was exposed and carefully resected. Full decompression was confirmed when osteophyte and herniated discs were removed under the pedicle and to the edge of the sacral alar (Fig. 5). Bleeding control was completely performed after stopping the irrigation. Then, a drainage tube was inserted, the arthroscope and instruments were removed, and the operation was finished after suturing the wounds. The operation time was 70 minutes, and blood loss was minimal. The leg pain and weakness improved dramatically, NIC improved to 50 minutes, and the modified MacNab criteria score was graded as good without surgical complications. Postoperative CT and MRI demonstrated that the foraminal stenosis was completely decompressed.

## 4. Results

Following the operations, we obtained clinical data for all the patients. VAS, Oswestry disability index and NIC assessments indicated significant improvements in pain and disability, all the pre- and postoperative clinical and radiologic data are summarized in Table 1.

**Table 1:** Cases with UBE revision for ASD.

Case	Age	Gender	Affected level	Pre-leg pain (VAS)	Pre-ODI	Pre-NIC (min)	Operation time	Post-leg pain (VAS)	Post-ODI	Post-NIC (min)	Hospital stays (d)	Patient satisfaction
1	56	Male	L3/4	3	50	25	45	1	12	60	3	Excellent
2	78	Female	L3/4	6	65	10	75	2	18	60	4	Excellent
3	67	Female	L5/S1	7	48	15	70	2	16	50	4	Good

ASD = adjacent segmental disease, NIC = neurological intermittent claudication, ODI = Oswestry disability index, UBE = unilateral biportal endoscopy, VAS = visual analog scale.

## 5. Discussion

Revision surgery is always a difficult procedure due to the scar tissue and previous instrumentation at the operative area. Full endoscopic revision surgery has been much less reported because of its inherent shortcomings, and the most frequent way to treat ASD is posterior laminectomy with extended fusion.^[14–16]^ As a new endoscopic technique, UBE has been applied in revision surgery. Choi^[17]^ has described the UBE revision details for recurrent LDH and recommended it as a useful technique for this entity. A recent study^[18]^ comparing the UBE (16 patients) *vs* microscopic (20 patients) revision for recurrent LDH has reported that UBE revision showed faster pain relief and earlier functional recovery, although both techniques showed good clinical outcomes. Kim^[19]^ has reported a satisfactory result using the same technique to remove the bony fragment immediately after minimally invasive techniques for transforaminal lumbar interbody fusion. Therefore, we assume that UBE should theoretically have better clinical outcomes in ASD with less scar tissue.

Lumbar stenosis caused by spinal epidural lipomatosis is rare, and an operation should be considered in patients whose symptoms are intractable to conservative treatment. Heo,^[20]^ in a study with 3 groups (biportal endoscopy, uniportal endoscopy, and microsurgery) with at least 1 year of follow-up, has reported that UBE showed benefits in terms of avoiding postoperative facet joint violation and reduced immediate postoperative pain. Other studies^[21–25]^ have also drawn attention to the advantages of a shorter hospitalization period and less postoperative back pain when compared with microscopic decompression. In our case, the epidural fat stuck with the dura mater was carefully removed with pituitary forceps, and full decompression was confirmed when the medial walls of bilateral pedicles were visualized. The clinical and radiologic improvements were achieved without durotomy, and the patient was discharged 48 hours after operation.

Due to the characteristics of upper lumbar facet joints, such as the narrow isthmus and sagittal articular surface, treating upper LDH via the UBE ipsilateral approach tends to cause damage to the isthmus and facet joints, leading to lumbar instability.^[26,27]^ The UBE technique with an ipsilateral approach can hardly allow the decompression of nerve roots at 2 levels (1 nerve root at the same level and the other at the cranial level in its foraminal area). Based on the above-mentioned anatomical factors, the contralateral sublaminar approach can better facilitate the removal of an up-migrated herniated disc to achieve the purpose of 2 nerve root decompression.^[28]^ In case 2, the up-migrated disc herniation was successfully removed without prolonged instrumentation. The design of the portals is very important. It is only when the portals deviate to the caudal side that the surgical tools can reach the target area to remove the up-migrated fragments.

Several reports^[29–32]^ have demonstrated that mono-portal endoscopic foraminoplasty is an effective and safe treatment for patients with lumbar foraminal stenosis. However, in patients with protruding iliac crest and hypertrophic ala, foraminoplasty at the L5 to S1 is quite challenging.^[33]^ In 2018, Kim^[34]^ suggested that UBE decompression with a 30° arthroscope was a minimally invasive surgery for decompressing L5 to S1 foraminal stenosis, preserving facet stability and providing symptomatic improvement. Others have also found that foraminal decompression using the UBE technique in various foraminal stenosis can preserve the motion of the L5 to S1 facet joint and decrease the need for fusion surgery.^[35–37]^ In case 3, a highspeed diamond burr could not only remove the metal rod occupying the working space but also drill the hypertrophic osteophyte in the foraminal area. Additionally, sufficient decompression of the L5 root was confirmed after the pulsation was observed, and the patient achieved full symptomatic relief.

In patients with unstable ASD, UBE-guided revision arthrodesis has been reported by Wang.^[38]^ The authors successfully performed a revision surgery for ASD with rigid fixation, and the patient received early postoperative ambulation without developing complications in the postoperative period.

There were some limitations of this study, such as the small sample size and short follow-up period. ASD is heterogeneous, and each patient has unique characteristics, requiring personalized surgical strategies from doctors. This study must strictly be interpreted as describing an alternative method for a selected group of ASD patients. Endoscopic surgery has a steep learning curve, and UBE revision surgery for ASD is even more complex, surgeons must undergo repeated training to master endoscopic surgery before they can perform it.

## 6. Conclusion

In summary, UBE allows for clearer and more sufficient visualization because of continuous irrigation and permits wider decompression due to multiple approaches; thus, it will play a role in various ASDs.

## Author contributions

**Conceptualization:** Hao Pan.**Data curation:** Susu Sun, Wei Zhang.

**Funding acquisition:** Wei Zhang.

**Investigation:** Chengyue Zhu, Yujun Zhang, Rongxue Shao, Jiaming Liang.

**Methodology:** Jiaming Liang.

**Project administration:** Chengyue Zhu, Jiaming Liang.

**Resources:** Jiaming Liang, Wei Cheng.

**Software:** Jiaming Liang.

**Supervision:** Hao Pan, Wei Zhang.

**Validation:** Wei Zhang.

**Visualization:** Jiaming Liang.

**Writing – original draft:** Chengyue Zhu.

**Writing – review & editing:** Chengyue Zhu.

## Supplementary Material

## References

[1] Zhang C Berven SH Fortin M. Adjacent segment degeneration versus disease after lumbar spine fusion for degenerative pathology: a systematic review with meta-analysis of the literature. Clin Spine Surg. 2016;29:21–9. 26836484 10.1097/BSD.0000000000000328

[2] Anandjiwala J Seo JY Ha KY. Adjacent segment degeneration after instrumented posterolateral lumbar fusion: a prospective cohort study with a minimum five-year follow-up. Eur Spine J. 2011;20:1951–60. 21786038 10.1007/s00586-011-1917-0 PMC3207344

[3] Radcliff KE Kepler CK Jakoi A. Adjacent segment disease in the lumbar spine following different treatment interventions. Spine J. 2013;13:1339–49. 23773433 10.1016/j.spinee.2013.03.020

[4] Choi KC Kim JS Shim HK. Changes in the adjacent segment 10 years after anterior lumbar interbody fusion for low-grade isthmic spondylolisthesis. Clin Orthop Relat Res. 2014;472:1845–54. 23990447 10.1007/s11999-013-3256-4 PMC4016462

[5] Ghiselli G Wang JC Bhatia NN. Adjacent segment degeneration in the lumbar spine. J Bone Joint Surg Am. 2004;86:1497–503. 15252099 10.2106/00004623-200407000-00020

[6] Adogwa O Owens R Karikari I. Revision lumbar surgery in elderly patients with symptomatic pseudarthrosis, adjacent-segment disease, or same-level recurrent stenosis. Part 2. A cost-effectiveness analysis: clinical article. J Neurosurg Spine. 2013;18:147–53. 23231358 10.3171/2012.11.SPINE12226

[7] Ryu DS Park JY Kuh SU. Surgical outcomes after segmental limited surgery for adjacent segment disease: the consequences of makeshift surgery. World Neurosurg. 2018;110:e258–65. 29109064 10.1016/j.wneu.2017.10.150

[8] Drysch A Ajiboye RM Sharma A. Effectiveness of reoperations for adjacent segment disease following lumbar spinal fusion. Orthopedics. 2018;41:e161–7. 28662247 10.3928/01477447-20170621-02

[9] McGrath LB Jr Madhavan K Chieng LO. Early experience with endoscopic revision of lumbar spinal fusions. Neurosurg Focus. 2016;40:E10. 10.3171/2015.10.FOCUS15503 26828879

[10] Li T Zhu B Liu X. Revision strategy of symptomatic lumbar adjacent segment degeneration: full endoscopic decompression versus extended posterior interbody fusion. World Neurosurg. 2020;142:e215–22. 32599194 10.1016/j.wneu.2020.06.168

[11] Soliman HM. Irrigation endoscopic discectomy: a novel percutaneous approach for lumbar disc prolapse. Eur Spine J. 2013;22:1037–44. 23392557 10.1007/s00586-013-2701-0 PMC3657046

[12] Eun SS Eum JH Lee SH. Biportal endoscopic lumbar decompression for lumbar disk herniation and spinal canal stenosis: a technical note. J Neurol Surg A Cent Eur Neurosurg. 2017;78:390–6. 27652804 10.1055/s-0036-1592157

[13] Merter A Karaeminogullari O Shibayama M. Comparison of radiation exposure among 3 different endoscopic discectomy techniques for lumbar disc herniation. World Neurosurg. 2020;139:e572–9. 32330613 10.1016/j.wneu.2020.04.079

[14] Parker SL Shau DN Mendenhall SK. Factors influencing 2-year health care costs in patients undergoing revision lumbar fusion procedures. J Neurosurg Spine. 2012;16:323–8. 22284228 10.3171/2011.12.SPINE11750

[15] Jian W Yue Z Zheng FZ. Minimally invasive or open transforaminal lumbar interbody fusion as revision surgery for patients previously treated by open discectomy and decompression of the lumbar spine. Eur Spine J. 2011;20:623–8. 20927557 10.1007/s00586-010-1578-4 PMC3065602

[16] Smorgick Y Baker KC Fischgrund JS. Hidden blood loss during posterior spine fusion surgery. Spine J. 2015;15:2114–5. 26303182 10.1016/j.spinee.2015.06.021

[17] Choi DJ Jung JT Lee SJ. Biportal endoscopic spinal surgery for recurrent lumbar disc herniations. Clin Orthop Surg. 2016;8:325–9. 27583117 10.4055/cios.2016.8.3.325 PMC4987318

[18] Kang MS Hwang JH Choi DJ. Clinical outcome of biportal endoscopic revisional lumbar discectomy for recurrent lumbar disc herniation. J Orthop Surg Res. 2020;15:557. 33228753 10.1186/s13018-020-02087-6 PMC7685633

[19] Kim KR Park JY. The technical feasibility of unilateral biportal endoscopic decompression for the unpredicted complication following minimally invasive transforaminal lumbar interbody fusion: case report. Neurospine. 2020;17(Suppl 1):S154–9. 32746529 10.14245/ns.2040174.087 PMC7410383

[20] Heo DH Lee DC Park CK. Comparative analysis of three types of minimally invasive decompressive surgery for lumbar central stenosis: biportal endoscopy, uniportal endoscopy, and microsurgery. Neurosurg Focus. 2019;46:E9. 10.3171/2019.2.FOCUS197 31042664

[21] Chen T Zhou G Chen Z. Biportal endoscopic decompression vs. microscopic decompression for lumbar canal stenosis: a systematic review and meta-analysis. Exp Ther Med. 2020;20:2743–51. 32765769 10.3892/etm.2020.9001 PMC7401848

[22] Min WK Kim JE Choi DJ. Clinical and radiological outcomes between biportal endoscopic decompression and microscopic decompression in lumbar spinal stenosis. J Orthop Sci. 2020;25:371–8. 31255456 10.1016/j.jos.2019.05.022

[23] Kang KB Shin YS Seo EM. Endoscopic spinal surgery (BESS and UESS) versus microscopic surgery in lumbar spinal stenosis: systematic review and meta-analysis. Global Spine J. 2022;12:1943–55. 35333105 10.1177/21925682221083271 PMC9609515

[24] Lin GX Yao ZK Xin C. A meta-analysis of clinical effects of microscopic unilateral laminectomy bilateral decompression (ULBD) versus biportal endoscopic ULBD for lumbar canal stenosis. Front Surg. 2022;9:1002100. 36211279 10.3389/fsurg.2022.1002100 PMC9537863

[25] Park SM Park J Jang HS. Biportal endoscopic versus microscopic lumbar decompressive laminectomy in patients with spinal stenosis: a randomized controlled trial. Spine J. 2020;20:156–65. 31542473 10.1016/j.spinee.2019.09.015

[26] Akbary K Kim JS Park CW. Biportal endoscopic decompression of exiting and traversing nerve roots through a single interlaminar window using a contralateral approach: technical feasibilities and morphometric changes of the lumbar canal and foramen. World Neurosurg. 2018;117:153–61. 29857220 10.1016/j.wneu.2018.05.111

[27] Park JH Jang JW Park WM. Contralateral keyhole biportal endoscopic surgery for ruptured lumbar herniated disc: a technical feasibility and early clinical outcomes. Neurospine. 2020;17(Suppl 1):S110–9. 32746524 10.14245/ns.2040224.112 PMC7410376

[28] Kim JY Heo DH. Contralateral sublaminar approach for decompression of the combined lateral recess, foraminal, and extraforaminal lesions using biportal endoscopy: a technical report. Acta Neurochir (Wien). 2021;163:2783–7. 34436689 10.1007/s00701-021-04978-x

[29] Liu Y Van Isseldyk F Kotheeranurak V. Transforaminal endoscopic decompression for foraminal stenosis: single-arm meta-analysis and systematic review. World Neurosurg. 2022;168:381–91. 36527217 10.1016/j.wneu.2022.04.087

[30] Ahn Y Park HB Yoo BR. Endoscopic lumbar foraminotomy for foraminal stenosis in stable spondylolisthesis. Front Surg. 2022;9:1042184. 36439521 10.3389/fsurg.2022.1042184 PMC9687795

[31] Rhee DY Ahn Y. Full-endoscopic lumbar foraminotomy for foraminal stenosis in spondylolisthesis: two-year follow-up results. Diagnostics (Basel). 2022;12:3152. 36553159 10.3390/diagnostics12123152 PMC9777364

[32] Lin YP Wang SL Hu WX. Percutaneous full-endoscopic lumbar foraminoplasty and decompression by using a visualization reamer for lumbar lateral recess and foraminal stenosis in elderly patients. World Neurosurg. 2020;136:e83–9. 31866456 10.1016/j.wneu.2019.10.123

[33] Ahn Y Oh HK Kim H. Percutaneous endoscopic lumbar foraminotomy: an advanced surgical technique and clinical outcomes. Neurosurgery. 2014;75:124–33. 24691470 10.1227/NEU.0000000000000361 PMC4086756

[34] Kim JE Choi DJ. Unilateral biportal endoscopic spinal surgery using a 30° arthroscope for L5-S1 foraminal decompression. Clin Orthop Surg. 2018;10:508–12. 30505421 10.4055/cios.2018.10.4.508 PMC6250961

[35] Choi DJ Kim JE Jung JT. Biportal endoscopic spine surgery for various foraminal lesions at the lumbosacral lesion. Asian Spine J. 2018;12:569–73. 29879787 10.4184/asj.2018.12.3.569 PMC6002165

[36] Gandham EJ Uvaraj NR Eum JH. Unilateral biportal percutaneous transforaminal endoscopic lumbar foraminal decompression and discectomy: a technical note. Neurol India. 2022;70:510–4. 35532611 10.4103/0028-3886.344669

[37] Kim JE Choi DJ Park EJ. Clinical and radiological outcomes of foraminal decompression using unilateral biportal endoscopic spine surgery for lumbar foraminal stenosis. Clin Orthop Surg. 2018;10:439–47. 30505412 10.4055/cios.2018.10.4.439 PMC6250968

[38] Wang W Lyu P Liu Z. How I do it: biportal endoscopic spinal surgery for revision of adjacent segment disease after instrumented lumbar fusion. Acta Neurochir (Wien). 2022;164:2337–42. 35882672 10.1007/s00701-022-05318-3

